# Perioperative Kardioprotektion – „From bench to bedside“: Aktuelle experimentelle Evidenz und mögliche Gründe für die limitierte Translation in die klinische Praxis

**DOI:** 10.1007/s00101-020-00912-5

**Published:** 2021-01-19

**Authors:** Carolin Torregroza, Sebastian Roth, Katharina Feige, Giovanna Lurati Buse, Markus W. Hollmann, Ragnar Huhn

**Affiliations:** 1 Klinik für Anästhesiologie, Universitätsklinikum Düsseldorf, Moorenstr. 5, 40225 Düsseldorf, Deutschland; 2 Klinik für Anästhesiologie, Universitätsklinikum Amsterdam, Meibergdreef 9, 1105 AZ Amsterdam, Niederlande

**Keywords:** Pharmakologische Konditionierung, Präkonditionierung, Postkonditionierung, Ischämie-Reperfusion-Schaden, Herzchirurgie, Pharmacological conditioning, Preconditioning, Postconditioning, Ischemia-Reperfusion-Injury, Cardiac Surgery

## Abstract

**Hintergrund:**

Ziel der perioperativen Kardioprotektion ist es, die Auswirkungen eines Ischämie- und Reperfusionsschadens zu minimieren. Aus anästhesiologischer Sicht spielt dieser Aspekt insbesondere in der Herzchirurgie bei Patienten mit Einsatz der Herz-Lungen-Maschine, aber auch allgemein bei längerfristigen hypotensiven Phasen oder perioperativen ischämischen Ereignissen im nichtkardiochirurgischen Setting eine wichtige Rolle. Im Laufe der letzten Jahre konnten diverse pharmakologische sowie nichtpharmakologische Strategien der Kardioprotektion identifiziert werden. Die Ergebnisse von Studien an isoliertem Gewebe sowie von tierexperimentellen In-vivo-Studien sind vielversprechend. Eine Translation dieser kardioprotektiven Strategien in die klinische Praxis ist bislang jedoch nicht gelungen. Große klinische Studien konnten keine signifikante Verbesserung des Outcome der Patienten zeigen.

**Ziel der Arbeit:**

Dieser Übersichtsartikel gibt einen Überblick über die aktuelle experimentelle Evidenz pharmakologischer und nichtpharmakologischer Kardioprotektion. Außerdem sollen mögliche Gründe für die limitierte Translation diskutiert werden. Schließlich werden Möglichkeiten aufgezeigt, wie der Schritt „from bench to bedside“ in Zukunft doch noch gelingen könnte.

**Material und Methoden:**

Narrative Übersichtsarbeit.

**Ergebnisse und Diskussion:**

Trotz der vielversprechenden präklinischen experimentellen Ansätze zum Thema Kardioprotektion besteht nach wie vor eine große Diskrepanz zu den Ergebnissen aus großen klinischen Studien in der perioperativen Phase. Mögliche Gründe für die limitierte Translation könnten insbesondere Komorbiditäten und Komedikationen, die Wahl des Anästhesieverfahrens, aber auch die Wahl des Studiendesigns sein. Eine sorgfältige Studienplanung mit Berücksichtigung der genannten Probleme sowie ein simultaner Einsatz mehrerer kardioprotektiver Strategien mit dem Ziel eines additiven bzw. synergistischen Effekts stellen mögliche Ansätze für die Zukunft dar.

## Abstract (en)

**Background:**

Perioperative cardioprotection aims to minimize the consequences of myocardial ischemia reperfusion injury. These strategies appear particularly relevant for anesthesia provision during on-pump cardiac surgery but they potentially affect any cause of perioperative myocardial ischemia. In recent years, several pharmacological and nonpharmacological strategies of cardioprotection have been explored. Results from studies in isolated tissue and animal experiments are promising; however, translation of myocardial conditioning strategies to the clinical setting has been disappointing: in large trials cardioprotective interventions failed to significantly improve outcome.

**Objective:**

This review aims to provide an overview of the current experimental evidence regarding pharmacological and nonpharmacological cardioprotection. Moreover, it discusses reasons why translation from bench to bedside is hampered by potential confounders and suggests future approaches that might overcome these limitations.

**Material and methods:**

Narrative review.

**Results and conclusion:**

Results of experimental studies are convincing but translation into clinical practice remains challenging. Several confounders have been identified contributing to the mainly inconclusive results from clinical studies, such as comorbidities and comedications, choice of anesthetic regimen and also methodological issues. Carefully designed clinical trials in well-defined patient cohorts evaluating combinations of protective strategies targeting different pathways and cell types might support bench to bedside translation.

## Hinführung zum Thema

Kardiovaskuläre Erkrankungen stellen weltweit eine der häufigsten Todesursachen dar und sind mit einer signifikanten Einschränkung der Lebensqualität assoziiert. Eine Alterung der Bevölkerung sowie die Zunahme an kardiovaskulären Begleiterkrankungen führen auch im operativen Setting zu einer steigenden Anzahl an Patienten mit erhöhtem kardialen Risiko. Die perioperative Kardioprotektion ist dabei unabdingbar für eine optimale Versorgung dieser Patienten. Eine wichtige Herausforderung aus anästhesiologischer Sicht ist u. a. die Minimierung der Auswirkungen eines Ischämie- und Reperfusionsschadens bei Patienten mit Einsatz der Herz-Lungen-Maschine in der Herzchirurgie. Aber auch bei längerfristigen hypotensiven Phasen oder perioperativen ischämischen Ereignissen im nichtkardiochirurgischen Setting ist die Vermeidung bzw. Reduktion eines perioperativen Myokardschadens essenziell für das Patienten-Outcome. Im Laufe der letzten Jahrzehnte konnten diverse pharmakologische sowie nichtpharmakologische Strategien der Kardioprotektion identifiziert werden. Die Ergebnisse von Studien an isoliertem Gewebe sowie von tierexperimentellen In-vivo-Studien sind vielversprechend. Eine Translation dieser kardioprotektiven Strategien in die klinische Praxis ist bislang jedoch nicht gelungen. Große klinische Studien konnten keine signifikante Verbesserung des Outcome zeigen. In diesem Übersichtsartikel wird ein Überblick über die aktuelle experimentelle Evidenz pharmakologischer und nichtpharmakologischer Kardioprotektion gegeben. Dabei sollen nicht nur die einzelnen kardioprotektiven Strategien näher beleuchtet werden, sondern es werden auch mögliche Gründe für eine limitierte Translation diskutiert. Außerdem werden Möglichkeiten aufgezeigt, wie der Schritt „from bench to bedside“ in Zukunft vielleicht doch noch gelingen kann.

## Grundprinzipien der perioperativen Kardioprotektion

### Ischämie- und Reperfusionsschaden

Die zeitnahe Wiederherstellung der Durchblutung nach stattgefundener Ischämie stellt mit die wichtigste Behandlungsstrategie im klinischen Alltag dar, um das Ausmaß eines myokardialen Infarktareals zu begrenzen, und ist damit entscheidend für die Mortalität und Morbidität der Patienten. Paradoxerweise führt die Reperfusion nach einer Ischämie jedoch auch zu einem Schaden und Untergang von Kardiomyozyten, ist mitverantwortlich für bis zu 50 % der finalen Infarktgröße und mindert somit den vorteilhaften Effekt der wiederhergestellten koronaren Perfusion [1]. Dieser zusätzliche Gewebeschaden wird als **Ischämie- und Reperfusionsschaden (I/R-Schaden)** bezeichnet und beruht auf dynamischen zellulären Prozessen, hervorgerufen durch die mangelnde Sauerstoffversorgung in den Kardiomyozyten.

**Figure 1:**
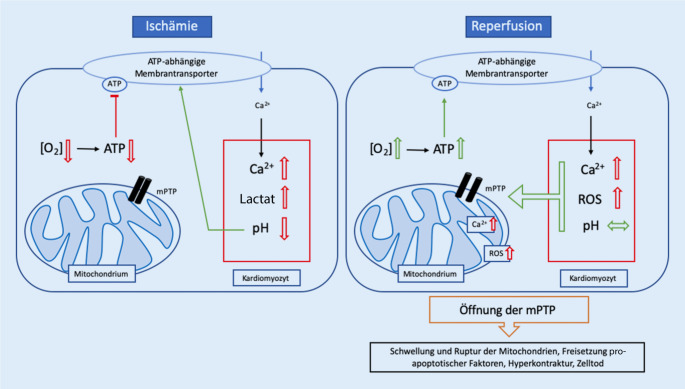

In Kürze zusammengefasst (Abb. 1) führen die anaeroben Bedingungen zu einem Mangel an Adenosintriphosphat (ATP) – und damit zur Inhibition ATP-abhängiger Membrantransporter – und einer Akkumulation von Lactat, was wiederum zu einer Reduktion des pH-Werts unter 7,0 führt [2]. Um diese Acidose auszugleichen, werden Protonen (H^+^) im Austausch gegen Natrium aus der Zelle heraustransportiert. Die daraus resultierende Natriumakkumulation in der Zelle induziert wiederum eine Umkehr des Natrium-Kalzium-Antiporters, wodurch zwar Natrium aus der Zelle eliminiert wird, die Kalziumkonzentration intrazellulär jedoch ansteigt. Durch die Reperfusion und das Wiederangebot von Sauerstoff kann der pH-Wert – durch die wiedereinsetzenden aeroben Prozesse – nahezu unmittelbar wieder auf Normalwerte angeglichen werden. Diese rapide pH-Verschiebung sowie der am Ende der Ischämie vorliegende intrazelluläre Kalziumüberschuss und zuletzt auch die – durch den oxidativen Stress freigesetzten – reaktive Sauerstoffspezies (ROS) führen zu **einer Öffnung der mitochondrialen Permeabilität-Transition-Pore (mPTP) **[1, 2]. Bei der mPTP handelt es sich um eine nichtspezifische Pore zwischen der inneren und äußeren Mitochondrienmembran, welche den Einstrom von Molekülen bis zu einem Molekulargewicht von 1500 Da erlaubt. Eine verlängerte Öffnung der mPTP resultiert in einem Verlust des mitochondrialen Membranpotenzials. Den Molekülen folgend strömt zudem Wasser entlang des osmotischen Gradienten in das Mitochondrium, was eine Schwellung und letztendlich Ruptur der äußeren Mitochondrienmembran mit **Freisetzung proapoptotischer Faktoren** bewirkt [3, 4]. Zuletzt führt der intrazelluläre Kalziumüberschuss, welcher durch die Ruptur der Mitochondrien noch verstärkt wird, zu einer Hyperkontraktur der Kardiomyozyten und damit zu einer weiteren **Schädigung der Zellmembran** der betroffenen und benachbarten Zellen.

### Bedeutung von „reperfusion injury salvage kinase“ und „survivor activating factor enhancement“

**Figure 2:**
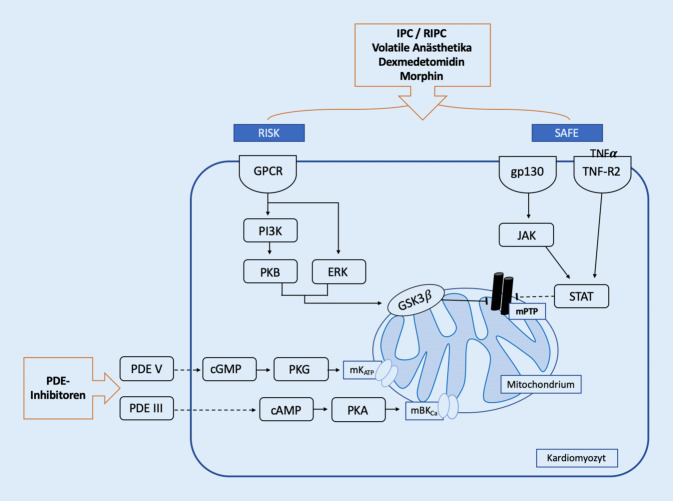

Auch wenn die grundlegenden Mechanismen der Kardioprotektion noch nicht abschließend im Detail geklärt sind, konnte bereits eine Vielzahl an beteiligten Signalwegen und Mediatoren identifiziert werden (Abb. 2; [6, 7]). An dieser Stelle sind insbesondere der ***Reperfusion-injury-salvage-kinase*(RISK)-** und ***Survivor-activating-factor-enhancement*(SAFE)**-Signalweg hervorzuheben, welche untereinander interagieren und das Mitochondrium als Endeffektor beeinflussen [5, 8, 9].

Vereinfacht zusammengefasst führt die Aktivierung des **RISK-Signalweges**, über G‑Protein-gekoppelte Rezeptoren, u. a. zu einer erhöhten Aktivität der Phospatidylinositol-3-Kinase (PI3K) sowie der nachgelagerten Proteinkinase B, Proteinkinase A und der „extracelullar-signal regulated kinases“ (ERK) [11]. Diese wiederum phosphorylieren die Proteinkinase C, endotheliale Stickstoffmonoxidsynthase (eNOS) und Glykogensynthasekinase-3 β (GSK3β). Phosphorylierte GSK3β liegt dadurch als inaktive Form vor, wodurch die Öffnung der mPTP inhibiert wird [7, 8].

Der **SAFE-Signalweg** beruht auf der Phosphorylierung von Januskinasen (JAK) über eine Bindung von Tumor-Nekrose-Faktor‑α (TNF-α) an die zugehörigen TNF-Typ-2-Rezeptoren [12]. Die JAK wiederum phosphoryliert und aktiviert „signal transducers and activators of transcription“ (STAT), wodurch ROS und die mPTP-Aktivität reguliert werden [13].

Aufgrund der Aktivierung dieser Signalwege kommt es zu einer verminderten **Öffnungswahrscheinlichkeit** der oben beschriebenen **mPTP** (Abb. 1), was folglich den durch Schwellung und Ruptur der Mitochondrienmembran sowie Freisetzung proapoptotischer Faktoren hervorgerufenen I/R-Schaden abschwächt [8, 14].

Neben der Regulierung der mPTP scheinen auch die **mitochondrialen Kaliumkanäle** im Kontext der Kardioprotektion entscheidend zu sein. In mehreren experimentellen Studien konnten hier insbesondere die **mitochondrialen ATP- und kalziumabhängigen Kaliumkanäle** identifiziert werden [15–17], welche durch Öffnung und damit Kaliumeinstrom zu einer Abnahme des mitochondrialen Membranpotenzials führen, was schließlich den Einstrom von Kalzium mindert und die Menge reaktiver Sauerstoffverbindungen reduziert [18]. Diese Mechanismen führen ebenfalls zu einer verringerten Öffnungswahrscheinlichkeit der mPTP und damit zur Protektion des Herzens gegen einen I/R-Schaden [3]. Paradoxerweise scheint im Kontext der Kardioprotektion bezüglich der Regulierung mitochondrialer Prozesse nicht das „Alles-oder-nichts“-Prinzip führend zu sein. So konnte gezeigt werden, dass eine gewisse Menge an ROS und auch eine transiente bzw. reversible Öffnung der mPTP essenziell sind, um eine Protektion der Kardiomyozyten zu erzielen. Lediglich die dauerhafte Aktivierung der mPTP und überschießende Mengen reaktiver Sauerstoffverbindungen scheinen schädlich zu sein [8].

## Experimentelle Evidenz perioperativer Kardioprotektion

### Nichtpharmakologische Kardioprotektion

#### Ischämische Präkonditionierung – Der „Ursprung“ der Kardioprotektion

Bereits 1986 konnte durch die Arbeitsgruppe von Murry et al. das Konzept **der ischämischen Kardioprotektion** („ischemic preconditioning“, IPC) im Hundemodell etabliert werden [19]. Unter IPC versteht man die Strategie, dass **kurze, subletale ischämische Episoden** – z. B. hervorgerufen durch Okklusion der Koronararterien – gefolgt von intermittierenden **Phasen der Reperfusion**, das Herz gegenüber einer darauffolgenden Ischämie und dem I/R-Schaden schützen können. Auch im translationalen Setting konnte bereits ein positiver Effekt von IPC im Rahmen von koronaren Bypass-Operationen nachgewiesen werden. So detektierten Yellon et al. 1993 einen erhöhten ATP-Gehalt in Myokardbiopsien nach IPC im Patienten, was sie auf eine verbesserte Geweberesistenz zurückführten [20]. Die zugrunde liegenden Mechanismen der IPC sind noch nicht abschließend geklärt; man geht aber davon aus, dass im Rahmen der subletalen Ischämien **endogene Stoffe (z.** **B. Adenosin, Bradykinin und Peptide)** freigesetzt werden [21], welche über G‑Protein-gekoppelte Rezeptoren intrazelluläre Signalwege – vornehmlich **RISK und SAFE** – aktivieren. Zudem führt IPC auch zu einer Aktivierung der mitochondrialen Kaliumkanäle und reguliert die ROS-Produktion – sodass letztendlich über die bereits oben beschriebenen Kaskaden die mPTP inhibiert und das Herz gegenüber einem I/R-Schaden geschützt wird [22, 23]. Die myokardiale Minderdurchblutung stellt in aller Regel – abgesehen vom operativen Setting – ein akutes und unvorhersehbares Ereignis dar, sodass die Präkonditionierung als Stimulus im klinischen Alltag impraktikabel erscheint. Um diese Problematik zu umgehen, wurden in den letzten Jahren insbesondere kardioprotektive Strategien erforscht, welche während der Ischämie (**Präkonditionierung**) oder der Reperfusion (**Postkonditionierung)** durchgeführt werden können. Neben der IPC führen auch subletale ischämische Episoden unmittelbar und bis zu 30 min nach Beginn der Reperfusion („ischemic postconditioning“, IPost) zu einer Kardioprotektion [24].

#### „Remote ischemic preconditioning“ – Die nichtinvasive Strategie

Während ischämische Konditionierungsmechanismen weiterhin als die effektivsten Strategien, das Herz in experimentellen Studien gegen einen I/R-Schaden zu schützen, beschrieben sind, bleibt die Anwendung von IPC im klinischen Setting aufgrund der hohen Invasivität wenig praktikabel. Interessanterweise können auch intermittierende Episoden von **subletaler Ischämie und Reperfusion in zielorganfernen Arealen** eine Organprotektion induzieren [25]. Dieses Phänomen wird als **ischämische Fernkonditionierung** („remote ischemic preconditioning“, RIPC) bezeichnet. RIPC ist dabei nicht auf ein bestimmtes Organ oder Gewebe als Stimulus beschränkt. So konnte beispielsweise sowohl für die Okklusion einer Mesenterial- als auch einer Nierenarterie eine Reduktion des myokardialen Infarktareales in tierexperimentellen Studien detektiert werden [26]. Das ideale RIPC-Protokoll – bezüglich Ort, Dauer, Anzahl und Intensität des Stimulus – ist zwar bisher noch nicht gänzlich identifiziert, jedoch haben sich insbesondere die Extremitäten (Oberarm oder Oberschenkel) als günstiges zielorganfernes Gewebe für RIPC etabliert. Dies ist nicht zuletzt zurückzuführen auf die geringe Invasivität und die hohe Praktikabilität des Stimulus – was wiederum eine leichtere Translation in das klinische Setting ermöglicht. Hierfür kann mittels einer Blutdruckmanschette, z. B. am Oberarm, der RIPC-Stimulus vermittelt werden. Auch im Rahmen von klinischen Studien zur Organprotektion wurde RIPC bereits untersucht [27]. Vergleichbar mit IPC, kann die ischämische Fernkonditionierung auch während der Ischämie oder der Reperfusion (RIPost) durchgeführt werden. Eine Vielzahl an humoralen Faktoren – Zytokine, Chemokine, NOS –, aber auch neuronale sowie vaskuläre Prozesse scheinen in die RIPC-vermittelte Kardioprotektion involviert zu sein [28]. Letztlich führt die Freisetzung der Botenstoffe am zielorganfernen Gewebe – durch Verteilung über den Blutkreislauf – zu einer Aktivierung der bekannten Signalkaskaden (**RISK** und **SAFE**) in den Kardiomyozyten [28].

### Pharmakologische Kardioprotektion

Die bekannten Signalwege der ischämischen Konditionierungsstrategien (IPC und RIPC) können auch durch Applikation **diverser pharmakologischer Substanzen** imitiert werden und so eine Protektion des Herzens gegen einen I/R-Schaden vermitteln [29]. Die pharmakologische Postkonditionierung ist dabei vermutlich die aussichtsreichste Strategie: **nichtinvasiv, praktikabel und problemlos nach Auftreten des ischämischen Ereignisses** durchführbar. Insbesondere Medikamente, die bereits im klinischen Alltag eingesetzt werden, scheinen hierbei vielversprechende Kandidaten für die erfolgreiche Translation von „bench to bedside“. Im Folgenden Abschnitt werden daher konkret die im *anästhesiologischen Alltag* relevanten Medikamente in den Fokus genommen.

#### Volatile Anästhetika – die Favoriten?

Neben ihrer Wirkung als Inhalationsnarkotika vermitteln die **volatilen Anästhetika** – Sevofluran, Desfluran und Isofluran – als **Prä- und Postkonditionierungsstimuli** auch kardioprotektive Effekte, indem sie zahlreiche intrazelluläre Signalkaskaden triggern [30, 31]. Neben den klassischen Mechanismen – u. a. RISK, SAFE, mitochondriale Kaliumkanäle und Modulierung der mPTP – konnten auch vaskuläre und posttranskriptionale Prozesse herausgearbeitet werden, über die volatile Anästhetika eine Protektion der Kardiomyozyten erzielen [32–35]. Ein maßgeblicher Unterschied zwischen tierexperimentellen und klinischen Studien ist das Vorliegen möglicher **„Störfaktoren“ wie Alter und Komorbiditäten**. Sowohl eine Myokardhypertrophie [36] als auch Hyperglykämie [31] und das Alter [37] können den positiven Effekt von z. B. Isofluran auf die Kardiomyozyten im tierexperimentellen Design aufheben. Da die Zielgruppe der kardioprotektiven Maßnahmen aufgrund des steigenden kardialen Risikoprofils insbesondere ältere Patienten umfasst, ist der Einfluss des **Alters** auf Konditionierungsmaßnahmen von großer Bedeutung. In diesem Zusammenhang muss u. a. die Rolle von **Autophagie und Mitophagie** beleuchtet werden. Mitophagie, als spezifische Form der Autophagozytose, bewirkt einen **Abbau von geschädigten Mitochondrien** und verhindert damit die verheerende Produktion und Freisetzung von ROS im Rahmen des I/R-Schadens [38]. Im Alter nimmt die Fähigkeit der Mitophagie in den Zellen ab, was ein relevanter Ansatzpunkt für myokardiale Konditionierungsstrategien sein könnte. Es zeigt sich hier jedoch eine schmale Gratwanderung: Kardioprotektive Effekte werden zwar über eine gesteigerte Autophagie induziert, eine übermäßige Steigerung scheint aber verheerend in Bezug auf die kardiale Funktion nach einem I/R-Schaden zu sein. Sowohl Isofluran als auch Sevofluran vermitteln ihre kardioprotektiven Eigenschaften, indem sie die, im Rahmen des I/R-Schadens, beeinträchtigten Prozesse der Autophagie wiederherstellen [39].

Zuletzt konnte in verschiedenen tierexperimentellen Studien gezeigt werden, dass die gleichzeitige Applikation von Sevofluran keinen Einfluss auf andere Konditionierungsstrategien hat, wie z. B. RIPC und Präkonditionierung mit Milrinon oder Levosimendan [40]. Eine Kombination von sevofluran- und intralipidinduzierter Postkonditionierung konnte sogar einen **additiven Effekt** auf die Protektion des Herzens gegenüber einem I/R-Schaden im Tiermodell zeigen [41]. **Sevofluran** kann also an sich als **Konditionierungsstimulus** eingesetzt werden und bietet zudem den Vorteil, neben der hämodynamischen Stabilität, als Inhalationsanästhetikum keinen negativen Effekt auf die **pharmakologische Kardioprotektion** durch andere Substanzen zu haben.

#### Propofol – ein Paradox?

Aufgrund der paradoxen Eigenschaften von Propofol auf unterschiedliche Konditionierungsstrategien scheint der Einfluss des Hypnotikums im Kontext der Kardioprotektion insbesondere aus anästhesiologischer Sicht von großer Bedeutung zu sein. Durch das widersprüchliche Verhalten gestaltet sich die Thematik Propofol und Kardioprotektion eher komplex und wirft weiterhin ungeklärte Fragen auf. Eine propofolinduzierte **Prä- und Postkonditionierung** konnte in tierexperimentellen Studien gezeigt werden und beruht letztlich auf den bekannten und oben beschriebenen Mechanismen – dem Abbau von freien Sauerstoffverbindungen, dem Kalziumüberschuss und der Öffnung der mPTP [42]. In den letzten Jahren konnten aber auch alternative Signalwege detektiert werden. So führt Propofol zu einer Infarktgrößenreduktion über die Inhibition von transmembranständigen „Transient-receptor-potential-vanilloid“(TRPV)-4-Kationenkanälen, was wiederum auch die Menge an intrazellulär vorliegendem Kalzium vermindert [43]. Zudem vermittelt Propofol seine organprotektive Wirkung über die Genregulation auf der posttranskriptionalen Ebene, in dem es die Expression nichtcodierender microRNA nach stattgefundenem I/R-Schaden reguliert [44]. Trotz der vielversprechenden kardioprotektiven Wirkung bei Einzelgabe von Propofol führt die Kombination mit dem Hypnotikum paradoxerweise zu einer **Aufhebung** der kardioprotektiven Eigenschaften anderer Konditionierungsstrategien [42]. So konnte in tierexperimentellen In-vivo- als auch in In-vitro-Translationsstudien mit humanem Plasma gezeigt werden, dass Propofol den Effekt von RIPC vollständig aufhebt [45–47]. Einhergehend damit werden auch die infarktgrößenreduzierenden Eigenschaften von pharmakologischen Konditionierungsstrategien – wie z. B. Milrinon und Levosimendan – bei gleichzeitiger Propofolgabe aufgehoben [40]. Das Phänomen der **paradoxen Wirkungsweise** von Propofol im Kontext der Kardioprotektion ist nicht abschließend geklärt und benötigt weitere Studien über zugrunde liegende Mechanismen; allerdings scheint die Menge freier reaktiver Sauerstoffspezies mitentscheidend zu sein [41].

#### Dexmedetomidin – mehr als nur ein Sedativum?

Der α_2_-Rezeptor-Agonist Dexmedetomidin – im klinischen Alltag als Sedativum oder zur Delirprophylaxe eingesetzt – vermittelt präklinisch in experimentellen Studien kardioprotektive Wirkungen als Prä- und Postkonditionierungsstimulus über den **RISK**- und **SAFE**-Signalweg, Aktivierung mitochondrialer Kaliumkanäle sowie Regulation von microRNA und Transkriptionsfaktoren [5, 48]. Dexmedetomidin zeigt aber wesentliche und bemerkenswerte Vorteile gegenüber anderen pharmakologischen Strategien der Kardioprotektion. Bei vielen Substanzen muss der Postkonditionierungsstimulus direkt zu Beginn der Reperfusion erfolgen, um eine organprotektive Wirkung zu erzielen. Interessanterweise ist die Infarktgrößenreduktion durch Dexmedetomidin vollkommen unabhängig von dem Zeitpunkt oder der Dauer der Gabe während der Reperfusion und beruht auf der Aktivierung mitochondrialer ATP- und/oder kalziumabhängiger Kaliumkanäle [49]. Diese Eigenschaft könnte individuellere und v. a. flexiblere Therapieoptionen in der Klinik ermöglichen. Ein weiteres Herausstellungsmerkmal ist die weiterbestehende Effektivität der dexmedetomidininduzierten Kardioprotektion, sogar unter Einfluss von Komorbiditäten. So konnte eine Postkonditionierung mit Dexmedetomidin bei Typ-2-Diabetes-Ratten eine Infarktgrößenreduktion hervorrufen [50], und auch als Präkonditionierungsstimulus kann Dexmedetomidin unter akuter Hyperglykämie seine protektiven Eigenschaften weiterhin vermitteln [51]. Auch eine endotheliale Dysfunktion, welche insbesondere bei älteren Patienten – und damit der vorrangigen Zielgruppe der Kardioprotektion – vorkommt, zeigte keinen Einfluss auf die durch Dexmedetomidin induzierte Protektion von Kardiomyozyten in einem I/R-Tiermodell [52]. Allerdings scheint Dexmedetomidin in Teilen – vergleichbar, jedoch nicht im gleichen Ausmaß wie Propofol – die Wirkung anderer pharmakologischer Konditionierungsstrategien zu beeinflussen. Die Präkonditionierung sowohl mit Milrinon als auch mit Mannitol wird durch eine gleichzeitige Gabe von Dexmedetomidin im tierexperimentellen In-vitro-Setting vollständig aufgehoben [40, 51].

#### Morphin – „old but gold“

Während Morphin im anästhesiologischen Alltag immer mehr durch die synthetisch hergestellten Opioide (Fentanyl, Sufentanil, Remifentanil) ersetzt wird, bleibt es im Kontext der Kardioprotektion weiterhin relevant und vielversprechend. Dabei werden die kardioprotektiven Eigenschaften über Bindung an die G‑Protein-gekoppelten Opioidrezeptoren und folglich Aktivierung oben beschriebener Signalkaskaden (**RISK **und **SAFE**, mitochondriale Kaliumkanäle, reaktive Sauerstoffspezies und microRNA) vermittelt [53–55]. An dieser Stelle ist die unterschiedliche Expression der Opioidrezeptoren im humanen Herzen unter gesunden und krankhaften Bedingungen von besonderem Interesse. So konnte tierexperimentell gezeigt werden, dass insbesondere die μ‑Opioid-Rezeptoren in ischämischen Kardiomyozyten deutlich hochreguliert sind, wohingegen sie im gesunden Herzgewebe nahezu fehlen [53, 56]. Daher erscheint nicht nur die Wahl der Substanz, sondern auch die Affinität zu den verschiedenen Opioidrezeptoren von großer Bedeutung im Kontext der pharmakologischen Konditionierungsstrategie. Zudem ist erwähnenswert, dass die remifentanilinduzierte Kardioprotektion sowohl bei Diabetes als auch unter akuter Hyperglykämie im Tiermodell vollständig aufgehoben wird – vermutlich über den gesteigerten oxidativen Stress und damit die Beeinträchtigung des RISK- und SAFE-Signalweges [57]. Morphin scheint dahingegen den Vorteil zu bieten, dass eine postischämische Behandlung nicht nur zu einer Abnahme der Infarktgröße, sondern auch zur Reduktion der myokardialen Dysfunktion, Fibrose und des Remodeling führt und damit die linksventrikuläre Funktion nach stattgefundenem Myokardinfarkt verbessert [58]. Dabei werden die kardioprotektiven Eigenschaften von Morphin, neben Aktivierung der oben genannten Signalwege, insbesondere über eine gesteigerte NO-Synthese vermittelt, wie sowohl in tierexperimentellen Studien als auch in Untersuchungen an humanem Gewebe gezeigt werden konnte [59]. Interessanterweise scheint auch das zentralnervöse System an der Vermittlung kardioprotektiver Effekte von Morphin beteiligt zu sein, vornehmlich über eine verminderte Expression vom „nerve growth factor“ und Aktivierung sensorischer Ionenkanäle in Nervenzellen [60]. Neben den bekannten Signalwegen der Kardiomyozyten scheinen also auch extrakardiale und extrazelluläre Prozesse im Kontext der Kardioprotektion von Bedeutung zu sein.

#### Phosphodiesterase-Inhibitoren – ein neuer Ansatz?

Im perioperativen Setting der Kardioanästhesie haben sich Phosphodiesterase (PDE)-Inhibitoren, wie Milrinon und Sildenafil, zur unterstützenden Therapie der Herzinsuffizienz und positiven Inotropie (PDE-III-Hemmer) sowie zur Behandlung einer akuten Rechtsherzbelastung (PDE-V-Hemmer) in den letzten Jahren zunehmend etabliert. In diesem Zusammenhang stellt sich also die Frage, ob auch ein protektiver Effekt an den Kardiomyozyten im Rahmen des I/R-Schadens hervorgerufen werden kann, was diesen Substanzen im klinischen Alltag noch mehr Bedeutung zukommen lassen würde. Auf Basis tierexperimenteller Studien konnte gezeigt werden, dass beide Gruppen der PDE-Inhibitoren zu einer Reduktion der Infarktgröße und Verbesserung der myokardialen Dysfunktion nach einer I/R führen [61–63]. Der **protektive Effekt **der PDE-V-Inhibitoren wird dabei über cGMP/PKG, bei den PDE-III-Inhibitoren über cAMP/PKA und anschließende intrazelluläre Signalkaskaden vermittelt. Die PDE-V-Hemmer scheinen dabei vornehmlich über mitochondriale ATP-abhängige Kaliumkanäle zu wirken, wohingegen PDE-III-Hemmer die mitochondrialen kalziumabhängigen Kaliumkanäle aktivieren. Schlussendlich wird über beide Signalwege die **Öffnungswahrscheinlichkeit der mPTP** reduziert [64, 65]. Aufgrund einer bekannten Beeinflussung zwischen cGMP und cAMP – und den nachgeschalteten Proteinkinasen – kann eine gegenseitige Wechselwirkung der PDE-Inhibitoren und ihrer Signalkaskaden jedoch nicht vollends ausgeschlossen werden. Interessanterweise vermittelt auch der Inodilator Levosimendan eine Inhibition der PDE III und führt über die Aktivierung der cAMP/PKA-Achse zu einer Protektion des Herzens gegenüber einem I/R-Schaden [66, 67]. Für die Translation in die Klinik ist jedoch zu beachten, dass sowohl Milrinon als auch Levosimendan durch das Anästhesieregime beeinflusst werden und so – in tierexperimentellen Studien – unter simultaner Propofolapplikation ihre kardioprotektiven Eigenschaften verlieren [40].

## Mögliche Gründe für die limitierte Translation in die klinische Praxis

Trotz der vielversprechenden Ergebnisse aus experimentellen Untersuchungen ist eine Translation kardioprotektiver Behandlungsmethoden in die klinische Praxis bislang nicht gelungen. Zwar konnten einzelne klinische Studien auch positive Effekte darlegen [68], ein eindeutiger Vorteil für Patienten konnte aber in den großen klinischen Outcome-Studien sowohl für pharmakologische [69–71] als auch für nichtpharmakologische [72–75] Therapien bisher noch nicht gezeigt werden. Daher beschäftigt sich dieser Übersichtsartikel nun primär mit möglichen Gründen für eine limitierte Translation. Hierbei sollen insbesondere der Einfluss von Komorbiditäten und Komedikationen, die Wahl des Anästhesieverfahrens sowie die Bedeutung des Studiendesigns bzw. die Relevanz einer methodologisch korrekten Vorgehensweise beleuchtet werden.

### Komorbiditäten und Komedikationen – eine individuelle Betrachtung

Die Unterschiede zwischen einer experimentellen Untersuchung und einer pragmatischen klinischen Studie sind offensichtlich sehr groß. Während die Versuche im Labor in der Regel unter klar definierten Bedingungen strikt nach Protokoll ablaufen, muss im klinischen Setting eine Vielzahl von Einflussgrößen berücksichtigt werden. Dazu zählen ganz besonders Komorbiditäten sowie Komedikationen, die potenziell zu einer Verfälschung der Ergebnisse führen können. Ein Schwerpunkt der Forschung der letzten Jahre wurde auf den Einfluss einer diabetischen Erkrankung sowie des Patientenalters gelegt [76, 77]. Bezüglich des Diabetes mellitus gibt es bereits Hinweise darauf, dass eine Hyperglykämie kardioprotektive Effekte negativ beeinflussen könnte. Wie bereits erwähnt, konnten tierexperimentelle Untersuchungen zeigen, dass sowohl RIPC als auch pharmakologische Strategien der Kardioprotektion in einem Rattenmodell mit Typ-2-Diabetes zu keiner signifikanten Reduktion der Infarktgröße führen [31, 76, 78, 79].

Im klinischen Setting existieren bisher nur wenige Studien, die sich mit der Frage beschäftigt haben, ob RIPC bzw. eine pharmakologische Konditionierung durch das Vorliegen eines Diabetes mellitus negativ beeinflusst werden könnte. So konnte eine retrospektive Studie beispielsweise zeigen, dass RIPC bei Diabetikern keinen positiven Effekt hatte und sich sogar negativ auswirkte, wenn Patienten Sulfonylharnstoffe in ihrer Medikation hatten [80]. Eine mögliche Erklärung für den negativen Effekt könnte der Einfluss von Sulfonylharnstoffen auf mitochondriale Kaliumkanäle sein. Dieser Aspekt wurde bereits in weiteren Studien zur ischämischen Konditionierung untersucht. So konnte eine Studie an humanem Gewebe des rechten Vorhofes zeigen, dass der Effekt von IPC durch die Langzeiteinnahme von Sulfonylharnstoffen blockiert wird. Eine weitere prospektive placebokontrollierte Studie mit einer ähnlichen Fragestellung unterscheidet zusätzlich zwischen den verschiedenen Wirkstoffen und kommt zu dem Schluss, dass der protektive Effekt von IPC durch die Einnahme von Glibenclamid blockiert wird, bei Einnahme von Glimepirid jedoch erhalten bleibt. Um den klinischen Einfluss von Diabetes mellitus auf pharmakologische und nichtpharmakologische kardioprotektive Therapien evidenzbasiert beurteilen zu können, bedarf es weiterer großer Studien mit translationalem Ansatz.

Im Hinblick auf das Patientenalter geht es u. a. um die Tatsache, dass die Alterung des Herzens zu strukturellen Veränderungen führt, die die Freisetzung der kardioprotektiv wirksamen humoralen Faktoren beeinflussen könnten [81]. Zudem haben ältere Patienten häufiger kardiovaskuläre Vorerkrankungen und nehmen damit begleitend Medikamente ein, wie z. B. Acetylsalicylsäure, Statine oder diverse Antihypertonika, die einen Einfluss auf Mechanismen der Kardioprotektion haben könnten [10, 82]. Im Hinblick auf die Interferenz zwischen Kardioprotektion und Komedikationen liegt derzeit die beste Evidenz für Thrombozytenaggregationshemmer vor. Diese scheinen selbst einen kardioprotektiven Effekt zu vermitteln, was möglicherweise limitierend ist, für den Nachweis eines zusätzlichen kardioprotektiven Effektes durch RIPC oder eine pharmakologische Konditionierung [76]. Bezüglich des Einflusses des Alters konnte eine prospektive, randomisierte Studie mit 80 Patienten zeigen, dass der kardioprotektive Effekt von RIPC bei Patienten über 68 Jahren abgeschwächt ist [83]. Eine weitere Studie kam zu dem Schluss, dass die myokardiale Ischämietoleranz bei jüngeren Patienten höher ist als bei Patienten über 65 Jahren, was ebenfalls dafür sprechen könnte, dass kardioprotektive Strategien bei dieser Patientengruppe weniger effektiv sind [84]. Ob ältere Patienten aus den genannten Gründen wirklich schlechter auf kardioprotektive Therapien ansprechen, kann bislang allerdings noch nicht abschließend beantwortet werden. Eine ausführlichere Übersicht zum Einfluss von Diabetes mellitus und fortgeschrittenem Alter auf Mechanismen der Kardioprotektion bieten weitere bereits veröffentlichte Übersichtsarbeiten [76, 77].

### Die Wahl des Anästhesieverfahrens – *ein aktuelles Thema*

Einen wichtigen Einflussfaktor, der v. a. im Hinblick auf die limitierte Translation im perioperativen Setting diskutiert wird, stellt die Wahl des Anästhesieverfahrens dar. Propofol scheint, basierend auf experimentellen Daten, mit kardioprotektiven Mechanismen zu interferieren [45, 46]. Interessant ist in diesem Zusammenhang v. a., dass 2 multizentrische, randomisierte kontrollierte Studien zum Effekt von RIPC bei herzchirurgischen Patienten jeweils eine propofolbasierte Anästhesie verwendet haben und beide keine Verbesserung des klinischen Outcome zeigen konnten [72, 75]. So kommt der ERICCA Trial von Hausenloy et al. in einer randomisierten kontrollierten Multizenterstudie mit 1612 Patienten zu dem Schluss, dass RIPC bei Patienten, die sich einer koronaren Bypass- bzw. einer Herzklappenoperation unterziehen, und bei denen die Aufrechterhaltung der Allgemeinanästhesie mit Propofol erfolgte, zu keiner Verbesserung des klinischen Outcome führt [72]. Ein ähnliches Ergebnis lieferte der deutsche RIPHeart Trial von Meybohm et al., der in 1403 herzchirurgischen Patienten mit propofolbasierter Anästhesie ebenfalls keinen relevanten Benefit von RIPC zeigen konnte [75]. Beide Studien verwendeten einen zusammengesetzten primären Endpunkt aus Mortalität und kardiovaskulären Komplikationen. Eine 2018 veröffentlichte Sekundäranalyse des RIPHeart Trial zeigte außerdem, dass RIPC in dieser Studie zu keiner Freisetzung der kardioprotektiven humoralen Faktoren führte, was die Hypothese eines negativen Einflusses von Propofol zusätzlich unterstreicht [85]. Die Wahl des Anästhesieverfahrens stellt daher aktuell einen der vielversprechendsten Ansatzpunkte bei der Planung zukünftiger Studien dar. Eine Allgemeinanästhesie könnte auch ohne den Einsatz von Propofol erfolgen und stattdessen alternative Hypnotika zur Einleitung und volatile Anästhetika zur Aufrechterhaltung der Anästhesie verwendet werden. Erwähnenswert ist jedoch, dass aktuell noch unklar ist, ob eine Einmalgabe von Propofol als Einleitungshypnotikum bereits Einfluss auf kardioprotektive Effekte nimmt oder lediglich die kontinuierliche Applikation in dieser Hinsicht von Bedeutung ist.

### Ischämie ist nicht gleich Ischämie

Die meisten klinischen Studien zum Thema Kardioprotektion wurden an Patienten mit akutem Myokardinfarkt (AMI) und Indikation zur Koronarangiographie durchgeführt. Hierbei handelt es sich also um Strategien der Postkonditionierung, da das ischämische Ereignis in der Vergangenheit liegt. Bei Patienten mit AMI hängt der kardioprotektive Effekt jedoch von vielen Faktoren ab, u. a. von der Größe und Lokalisation des Infarktes oder auch vom Zeitpunkt bzw. Resultat der Koronarangiographie. Im Gegensatz dazu handelt es sich im herzchirurgischen Setting um eine geplante, globale Ischämie, in der Regel mit Einsatz der Herz-Lungen-Maschine, sodass hier auch Strategien der Präkonditionierung eingesetzt werden können. Diesbezüglich muss aber bedacht werden, dass herzchirurgische Patienten häufig bereits präoperativ Angina-pectoris-Beschwerden hatten, was eine eigene Art von ischämischer Präkonditionierung darstellt [86, 87] oder z. B. mit Glyceroltrinitrat vorbehandelt wurden, einem Wirkstoff, dem ebenfalls kardioprotektive Effekte zugeschrieben werden [88]. Beides könnte die Wirkmechanismen einer kardioprotektiven Behandlung beeinflussen, und so bestehen auch hier mögliche Störfaktoren, die es bei der Interpretation der klinischen Daten zu berücksichtigen gilt.

### Wahl des Studiendesigns: Translation mit Hindernissen

Bezüglich der großen Diskrepanz zwischen experimentellen und klinischen Ergebnissen soll ein weiterer Aspekt hier noch einmal ganz besonders beleuchtet werden: die Wahl des Studiendesigns bzw. die methodologische Herangehensweise. Unter dem Aspekt, dass klinische Studien eher ein heterogenes Patientenkollektiv mit diversen möglichen Einflussfaktoren (Diabetes mellitus, Arteriosklerose, Alter, Komedikation) abbilden, sollten experimentelle Studien vermehrt auch auf die Betrachtung eben genau dieser Einflussgrößen ausgerichtet sein. Während experimentelle Studien am gesunden Tier die Basis bilden, um grundlegende Signalwege und Mechanismen zu verstehen, sollten im nächsten Schritt Tiermodelle durchgeführt werden, welche mögliche Einflüsse von Pathologien auf Kardioprotektion untersuchen. Diese Relevanz wird unterstrichen durch die bisher bestehenden experimentellen Studien (z. B. Einfluss von DM 2 auf ischämische und pharmakologische Kardioprotektion), welche bereits negative Einflüsse von einzelnen Pathologien auf Konditionierungsstrategien nachweisen konnten. Wenn man dann an dem Punkt angekommen ist, dass eine kardioprotektive Behandlung auf der Basis experimenteller Erkenntnisse am Patienten getestet werden soll, dann folgt klassischerweise zunächst eine sog. Proof-of-concept-Studie. Im Bereich der Kardioprotektion handelt es sich dabei in der Regel um eine Studie, die eine kurzfristige Reduktion der Infarktgröße, in der Regel definiert als reduzierte Freisetzung von Troponin, als primären Endpunkt untersucht. Auch hierbei müssen aber zwei wichtige Punkte bedacht werden: Nicht nur die kardioprotektive Behandlung selbst, sondern beispielsweise auch das unmittelbare chirurgische Trauma einer herzchirurgischen Operation beeinflusst das Ausmaß des I/R-Schadens und schließlich die Troponinfreisetzung. Unter dem Aspekt, dass die Troponinfreisetzung abhängig vom Operationsausmaß stark variieren kann [89], könnte der primäre Endpunkt möglicherweise verfälscht werden. Daher scheinen Biomarker den myokardialen Schaden nicht gleichermaßen sensitiv abzubilden, wie z. B. bildgebende Verfahren oder die Messung der Infarktgröße im tierexperimentellen präklinischen Setting [90]. Weiterhin ist es auch im Rahmen einer Proof-of-concept-Studie sinnvoll, die untersuchte Studienpopulation zunächst klar zu definieren, um möglichst nah an die Gegebenheiten einer experimentellen Voruntersuchung heranzukommen. Erst der letzte Schritt wäre dann die Konzeption einer pragmatischen klinischen Studie, die das langfristige Patienten-Outcome untersucht. Diese Reihenfolge wird nicht immer eingehalten und könnte ein weiterer relevanter Grund für die limitierte Translation präklinischer Daten in die Klinik sein. Dazu kommt die Tatsache, dass sehr viele verschiedene Endpunkte verwendet werden (z. B. Mortalität *vs.* Lebensqualität), und auch die Durchführung der entsprechenden Behandlungen erfolgt teilweise auf sehr unterschiedliche Art und Weise (z. B. bezüglich der Anzahl an RIPC-Zyklen oder der Dosierung eines Wirkstoffes). Das Resultat ist eine Flut an unterschiedlichsten Studien, die nur sehr schwer miteinander verglichen werden können.

## Ausblick: Was bringt die Zukunft?

Es stellt sich nun die Frage, was man konkret tun kann, damit die Translation der Kardioprotektion in die klinische Praxis doch noch gelingt. Hierzu möchten wir zwei Aspekte ganz besonders betonen:

1. Ein möglicher Fokus in der experimentellen Forschung könnte in Zukunft vermehrt auf Tiermodellen mit klinisch relevanten Komorbiditäten und Komedikationen sowie dem Einfluss unterschiedlicher Anästhesieverfahren liegen. In der klinischen Forschung wiederum sollten zunächst Proof-of-concept-Studien an gut definierten Populationen durchführt werden, bevor die fundierte Planung einer pragmatischen Outcome-Studie beginnt. Hierbei ist v. a. auf die Wahl eines geeigneten primären Endpunktes, eine genaue Fallzahlplanung sowie auf eine einheitliche Durchführung der untersuchten Intervention zu achten.
2. Experimentelle Studien konnten verschiedene kardiale, aber auch extrakardiale und extrazelluläre Signalwege und Mechanismen identifizieren, die bei der Vermittlung kardioprotektiver Effekte beteiligt sind. Auf dieser Basis stellt sich die Frage, ob eine Kombination mehrerer Konditionierungsstrategien, mit unterschiedlichen Wirkmechanismen, möglicherweise einen additiven Effekt erzielen könnte und so eine stärkere Kardioprotektion erreicht wird [91]. So konnte man beispielsweise mit RIPC oder Xenon die klassischen Signalkaskaden (RISK, SAFE, PKG) aktivieren und gleichzeitig eine Hypothermie zum Schutz vor Apoptose bzw. Nekrose durchführen [91–93]. Auch ein additiver Effekt durch die Kombination von Prä- und Postkonditionierungsstrategien könnte eine Option darstellen und wurde tierexperimentell bereits untersucht [94]. In tierexperimentellen Studien zeigen jedoch nicht alle Kombinationen einen additiven Effekt, und es kann noch nicht abschließend beantwortet werden, welche Kombinationen von Strategien bzw. Wirkmechanismen am vielversprechendsten sind. In jedem Fall scheint eine Kombinationstherapie ein denkbarer Ansatz zu sein, um den multiplen Komorbiditäten und Komedikationen eines multimorbiden Patienten besser Rechnung zu tragen.

Trotz dieser vielversprechenden Ansätze muss natürlich auch in Erwägung gezogen, dass die genannten Strategien der pharmakologischen sowie nichtpharmakologischen Kardioprotektion „wirklich“ nicht funktionieren und auch in einer optimal durchgeführten klinischen Studie mit Beachtung aller potenziellen Störfaktoren kein positiver Effekt nachweisbar ist. Bis dieser Schluss gezogen werden kann, sollten aber zunächst alle möglichen Gründe für die limitierte Translation ausgeschlossen werden, da perioperative Kardioprotektion derzeit noch ein zu hohes Potenzial besitzt, das Outcome der Patienten zu verbessern.

## Fazit für die Praxis

- Medikamente aus dem anästhesiologischen Alltag (volatile Anästhetika, Dexmedetomidin und Opioide) sowie nichtpharmakologische Strategien der Kardioprotektion (u. a. die ischämische Fernkonditionierung) vermitteln im experimentellen Setting vielversprechende kardioprotektive Effekte.
- Nach wie vor konnten diese Effekte im klinischen Setting nicht reproduziert werden.
- Mögliche Gründe für die limitierte Translation könnten insbesondere Komorbiditäten und Komedikationen, die Wahl des Anästhesieverfahrens sowie die Wahl des Studiendesigns sein, die in präklinischen Studien nicht berücksichtigt wurden.
- Zukünftige Studien sollen genannte Probleme berücksichtigen sowie den gleichzeitigen Einsatz mehrerer Strategien der Kardioprotektion in Erwägung ziehen, um potenziell additive bzw. synergistische Effekte zu erzielen.
